# Artificial Intelligence-Enhanced Spirometry in Primary Healthcare: Opportunities for Early Detection of Small Airway Dysfunction

**DOI:** 10.3390/healthcare14193133

**Published:** 2026-09-22

**Authors:** Nowaf Y. Alobaidi

**Affiliations:** 1 Department of Respiratory Therapy, College of Applied Medical Sciences, King Saud bin Abdulaziz University for Health Sciences, Alahsa 31982, Saudi Arabia; alobaidinowaf@gmail.com; 2 King Abdullah International Medical Research Center, Alahsa 31982, Saudi Arabia; 3 Department of Respiratory Care Services, King Abdulaziz Hospital, Ministry of National Guard—Health Affairs, Alahsa 31982, Saudi Arabia

**Keywords:** artificial intelligence, spirometry, primary care, small airway dysfunction, MMEF, FEF_25–75_%, COPD, early detection, machine learning, clinical decision support

## Abstract

Spirometry underuse and persistent limitations in its technical quality and interpretation in primary healthcare (PHC) contribute to delayed and inaccurate diagnosis of chronic obstructive pulmonary disease (COPD) and asthma. Artificial intelligence (AI) has recently demonstrated, at the randomised controlled trial level, that it can improve spirometry interpretation by primary care clinicians. Maximal mid-expiratory flow (MMEF), a spirometric measure routinely derived from every forced expiratory manoeuvre, has been associated with subsequent airflow obstruction and lung function decline, particularly when conventional spirometric indices remain preserved, suggesting that it may contribute to earlier recognition of small airway dysfunction. However, no existing review has examined whether AI could specifically address the challenge of MMEF interpretation, and the incremental value of MMEF within AI-assisted spirometry models remains unestablished. This narrative review synthesises current evidence on AI-assisted spirometry in PHC across three evidence domains, diagnostic classification, quality control, and computational small airway assessment, and examines the potential for integrating MMEF with conventional spirometric indices, flow–volume curve morphology, technical quality, clinical characteristics, and longitudinal data to support its clinical interpretation. The available evidence supports the feasibility of the individual components underpinning this proposed framework, including AI-assisted spirometry interpretation, automated quality assessment, and computational extraction of small airway information from spirometric data. However, the contextual integration of MMEF within AI-assisted models has not yet been prospectively evaluated, and validation of its incremental predictive value and clinical utility is needed before implementation can be recommended.

## 1. Introduction

Chronic obstructive pulmonary disease (COPD) and asthma together represent a major and growing burden of chronic disease worldwide [1,2]. Spirometry plays a central role in their diagnosis, yet pathological and physiological abnormalities may develop before conventional spirometric criteria become abnormal [3,4,5]. Primary healthcare (PHC) is often the first point of contact for patients presenting with respiratory symptoms, placing it in a key position to support earlier recognition and diagnosis of chronic respiratory diseases [6]. Yet spirometry in PHC remains underused and is subject to important limitations in technical quality and interpretation, contributing to diagnostic misclassification of chronic respiratory disease [7,8,9].

The clinical consequences of these gaps may be substantial. In a nationwide cohort of 146,205 patients initiating chronic respiratory treatment, spirometry performed at treatment initiation was associated with a 34% lower risk of all-cause mortality compared with no spirometry (adjusted HR 0.66, 95% CI 0.63–0.70) [10]. Diagnostic misclassification represents a further concern. A systematic review and meta-analysis found substantial diagnostic misclassification in PHC, with undiagnosed spirometry-confirmed COPD occurring in 14–26% of symptomatic smokers, while 25–50% of patients labelled as having COPD had no post-bronchodilator airflow obstruction when formally tested [11]. Current spirometric interpretation is anchored primarily in conventional parameters, including forced expiratory volume in one second (FEV_1_), forced vital capacity (FVC), and the FEV_1_/FVC ratio [12,13]. The interpretive uncertainty is greater for maximal mid-expiratory flow (MMEF; also reported as FEF_25–75_%), a routinely derived spirometric measure commonly considered an indirect marker of peripheral airway function [14], for which the clinical significance of an abnormal value and its implications for clinical decision-making remain uncertain [12].

Artificial intelligence (AI) has emerged as a promising approach to addressing the spirometry interpretation gap in PHC. The SPIRO-AID randomised controlled trial (RCT) demonstrated that AI decision support significantly improved spirometry interpretation by PHC clinicians compared with unassisted interpretation, including diagnosis prediction, pattern recognition, and technical quality assessment [15]. At the same time, a growing body of evidence suggests that reduced MMEF may provide information relevant to small airway dysfunction (SAD) before conventional spirometric obstruction becomes apparent and has been associated with subsequent airflow obstruction, accelerated lung function decline, and structural and physiological features of COPD [16,17,18,19].

Despite the growing evidence base for AI-assisted spirometry interpretation and the prognostic significance of reduced MMEF, no existing review has examined whether AI could specifically address the challenge of MMEF interpretation in PHC. The incremental value of MMEF within AI-assisted spirometry models, and the conditions under which an abnormal MMEF finding should influence clinical decision-making, remain unresolved. This review examines the evidence for AI-assisted spirometry interpretation in PHC, evaluates the potential for AI to enhance the clinical interpretation of MMEF and support earlier recognition of SAD, discusses the associated technical and implementation challenges, and identifies priorities for future research.

## 2. Methods

### 2.1. Review Design

This narrative review examines the current evidence for AI-assisted spirometry interpretation in PHC and evaluates the potential for contextual integration of MMEF within AI-assisted spirometry models to support its clinical interpretation. The narrative review approach was selected because the objective was to critically integrate evidence across heterogeneous study designs, AI methodologies, clinical populations, and outcome measures, and to examine the conceptual relationship between existing AI applications and the specific challenge of MMEF interpretation in primary care. The purpose was therefore interpretive and integrative synthesis rather than answering a narrowly defined intervention or diagnostic-effectiveness question or generating a pooled effect estimate.

### 2.2. Search Strategy

Searches were performed in PubMed/MEDLINE and Google Scholar with no restriction on publication date. No language restriction was applied, although all studies identified and included were published in English.

The following keyword groups were used and combined using Boolean operators:

- AI and computational methods: “artificial intelligence”, “machine learning”, “deep learning”, “neural network”, “random forest”, “convolutional neural network”, “explainable AI”;
- Spirometry and lung function: “spirometry”, “forced expiratory flow”, “pulmonary function test”, “flow-volume curve”, “spirometry quality”;
- Small airways and MMEF: “maximal mid-expiratory flow”, “FEF_25–75_”, “FEF_25–75_%”, “small airway dysfunction”, “small airways”; and
- Clinical context: “primary care”, “primary healthcare”, “general practice”, “clinical interpretation”, “decision support”.

Reference lists of retrieved articles and relevant systematic reviews were also checked to identify additional studies not captured by the database searches.

### 2.3. Study Selection

Studies were included if they applied AI or machine learning—using data-driven approaches rather than pre-specified rule-based systems—to spirometry data or related physiological measurements, including raw spirometric curves, flow–volume loop morphology, or impulse oscillometry, and reported quantitative performance outcomes. Studies addressing automated spirometry quality control against ATS/ERS criteria, computational extraction of information from expiratory curve morphology, or AI-assisted interpretation in primary care or general practice settings were also eligible.

Studies were excluded if they applied AI exclusively to treatment selection, disease monitoring, or outcome prediction without relevance to spirometry interpretation or small airway assessment, or if they did not use spirometry or related physiological measurements as primary model inputs. Conference abstracts and non-peer-reviewed publications without quantitative outcome data were also excluded.

### 2.4. Evidence Synthesis

For each included study, the following information was extracted: study design, clinical setting, AI methodology, input data type, primary task, key performance metrics, relevance to MMEF or small airway dysfunction, and whether validation in a primary care population was reported. Findings were organised across three evidence domains: AI for diagnostic classification of spirometry, AI for spirometry quality control, and computational approaches to small airway assessment. These three domains and inform the proposed framework for AI-enhanced MMEF interpretation discussed in Section 4. Risk of bias was not formally assessed as the objective was conceptual synthesis rather than effect estimation.

## 3. Spirometry in PHC and Artificial Intelligence: Current Evidence

### 3.1. Spirometry in PHC: The Quality and Interpretation Gap

Spirometry is the recommended standard for confirming the diagnosis of COPD and for supporting the diagnosis of asthma [20,21]. Despite this, its performance and interpretation in PHC settings remain problematic. An influential study from the United States of America found that only one of 17 spirometers tested in PHC offices met accuracy criteria, and that spirogram quality was acceptable for clinical use in only 60% of patients [8]. Technical errors during spirometry, including submaximal inhalation, variable effort, early termination, and coughing during the manoeuvre, can impair the reliability of the measurements obtained [12,13]. MMEF is especially susceptible to such technical limitations because it depends on the achieved FVC and may therefore be affected by incomplete or technically inadequate expiratory manoeuvres [12].

Beyond technical quality, accurate interpretation of spirometry remains a challenge in PHC. In a PHC study, 96.5% of spirometry traces were technically adequate for interpretation; however, among patients with a recorded COPD diagnosis, only 72.5% of acceptable traces showed airflow obstruction on independent assessment by respiratory physicians [7]. Similarly, Walters et al. found that spirometric confirmation of COPD was absent in 31% of 341 patients carrying a primary care diagnosis of COPD [22]. These findings are reflected at a broader level in a systematic review and meta-analysis, which found that 14–26% of symptomatic smokers attending PHC had spirometry-confirmed COPD that remained undiagnosed, while 25–50% of patients labelled as having COPD showed no post-bronchodilator airflow obstruction when formally tested [11].

The interpretive challenge is compounded by the complexity of current spirometry reporting. Reports may include multiple parameters, including FEV1, FVC, FEV1/FVC, MMEF, FEF50%, and FEF75%, although the clinical significance of abnormalities beyond the conventional indices is often uncertain. MMEF is a particularly relevant example because commonly used percentage-predicted thresholds can classify abnormality differently from reference-based z-scores. Almeshari et al. found that using an MMEF threshold of <80% predicted produced false-positive rates exceeding 24% compared with a z-score-based definition, with discordance influenced by demographic and anthropometric characteristics [23]. Thus, the challenge is not simply whether MMEF is reported but how an abnormal value should be interpreted in the context of conventional spirometric indices, technical quality, and patient characteristics. This represents a potential application for AI-assisted interpretation.

### 3.2. AI in Spirometry Interpretation: The Evidence Base

The application of AI to spirometry interpretation encompasses a range of computational approaches, each with distinct characteristics relevant to the tasks described in this section. Supervised machine learning algorithms are trained on labelled spirometry data to identify patterns and generate diagnostic predictions. Among these, random forest models build multiple decision trees from random subsets of spirometric features and aggregate their outputs, offering robust classification with an inherent measure of each feature’s contribution to the prediction. Explainable AI extends this further by providing interpretable explanations of model outputs (commonly through Shapley values), allowing clinicians to understand which spirometric features drove a particular classification. Deep learning approaches, including convolutional neural networks, apply learned filters directly to raw spirometric curves and flow–volume loop data, extracting complex structural patterns without requiring manual feature specification. Artificial neural networks more broadly learn non-linear relationships between spirometric inputs and diagnostic outputs through multiple processing layers. AdaBoost, an ensemble method, combines multiple classifiers sequentially and gives greater weight to previously misclassified cases, improving performance on diagnostically challenging spirometry records [24].

#### 3.2.1. Progress from Specialist to PHC Settings

The potential for AI to improve pulmonary function test interpretation was demonstrated by Topalovic and colleagues, who compared a machine-learning-based diagnostic system trained to identify disease patterns directly from spirometric and pulmonary function data with 120 pulmonologists from 16 hospitals across five European countries [25]. The AI system achieved a diagnostic accuracy of 82%, compared with 44.6% among pulmonologists (*p* < 0.0001) [25]. A subsequent study evaluated whether explainable AI, which provides interpretable explanations of model predictions using Shapley values allowing clinicians to understand the contribution of each spirometric feature to the classification output, could complement rather than replace specialist interpretation [26]. In the multicentre phase involving 62 pulmonologists, AI assistance increased preferential diagnostic accuracy from 69.3% to 74.6% and differential diagnostic accuracy from 81.7% to 90.4% (*p* < 0.0001) [26]. Notably, the combination of pulmonologist and AI also outperformed AI alone, supporting a complementary role for AI in pulmonary function test interpretation.

Translation to PHC settings presents additional challenges because spirometry performed in this setting may be of suboptimal technical quality and is typically available without the additional pulmonary function measurements used to develop earlier AI models. Sunjaya and colleagues, therefore, evaluated AI diagnostic support using retrospective spirometry data from 1113 patients attending PHC in northwest London [27]. Using a supervised random-forest model that constructs multiple decision trees from random subsets of spirometric and demographic features and aggregates their outputs to generate a diagnostic probability, the AI software identified COPD with a sensitivity of 84.0% (95% CI 80.6–87.0%), specificity of 86.8% (95% CI 83.8–89.5%), and overall accuracy of 85.4% (95% CI 83.2–87.5%), with an area under the receiver operating characteristic curve of 0.914 (95% CI 0.896–0.930). Discrimination was also demonstrated for interstitial lung disease and asthma, although performance was lower, with AUCs of 0.900 and 0.814, respectively [27]. These findings provide evidence that AI can support diagnostic interpretation of spirometry in PHC, particularly for COPD, using spirometry and basic demographic data without additional pulmonary function testing.

The most robust evidence to date comes from the SPIRO-AID RCT [15]. In this parallel-group superiority trial, 234 PHC clinicians were randomised to interpret 50 real-world patient spirometry records with or without AI decision support, with 133 clinicians completing all 50 cases. AI assistance improved preferred diagnosis prediction performance by 9.0 percentage points compared with unassisted interpretation (95% CI 4.5–13.3; *p* = 0.001), with additional improvements in differential diagnosis prediction, technical quality assessment, pattern interpretation, and self-rated confidence [15]. The effect was greater in the prespecified COPD subgroup, where AI assistance improved preferred diagnosis prediction performance by 15.9 percentage points (95% CI 9.0–22.7; *p* < 0.001). These findings provide randomised evidence that AI decision support can improve the interpretive performance of PHC clinicians, extending previous evidence of standalone AI diagnostic accuracy to demonstrate measurable benefit when AI is integrated into clinician interpretation.

Across the studies described above, validation in PHC settings has been limited. Of the studies examining AI for diagnostic classification, only Sunjaya et al. and the SPIRO-AID trial were conducted in or directly validated for PHC settings. The remaining diagnostic classification studies used specialist pulmonary function laboratory data, where spirometry quality is typically higher, additional measurements are available, and patient populations are more selected. Studies of AI quality control showed similarly limited PHC validation, with only the Wang et al. model tested in a general practitioner setting. Studies examining computational approaches to small airway detection were conducted exclusively in specialist or research cohorts. While the proof of concept for AI-assisted spirometry interpretation is therefore well established in specialist settings, translation to PHC, where spirometry quality is more variable, clinical information more limited, and the diagnostic challenge greatest, requires dedicated validation in PHC populations. This gap is particularly relevant to AI-enhanced MMEF interpretation, since the incremental value of MMEF may differ between specialist and PHC settings where the pre-test probability of disease and the quality of the spirometric manoeuvre vary substantially.

#### 3.2.2. AI for Spirometry Quality Control

An important application of AI in spirometry is automated quality control, which assesses whether individual manoeuvres meet American Thoracic Society/European Respiratory Society (ATS/ERS) standards for acceptability and usability [13]. Conventional quality assessment combines quantitative criteria with visual inspection of flow–volume and volume–time curves, which can be time-consuming and subject to inter-reviewer variability. Das and colleagues developed convolutional neural networks using 36,873 spirometry curves from the National Health and Nutrition Examination Survey [28]. In an independent test set of 3738 curves, the models achieved accuracies of 87% for acceptability and 92% for usability, with usability classification achieving 92% sensitivity and 96% specificity. The models significantly outperformed ATS/ERS quantitative rule-based assessment (*p* < 0.0001). Subsequent work extended automated quality assessment to both spirometric indices and curve morphology. Wang and colleagues developed a deep-learning model using 16,502 spirometry files [29]. In internal testing, the model achieved accuracies of 95.1%, 92.4%, and 94.3% for FEV_1_ acceptability, usability, and quality grading, respectively, with corresponding accuracies of 93.6%, 94.3%, and 92.2% for FVC. Importantly, implementation of the model was associated with increases of approximately 21% and 36% in the monthly proportion of good-quality FEV_1_ and FVC tests performed by general practitioners, respectively.

The scalability of automated quality control was subsequently examined in 21,795 spirometry sessions from the Canadian Longitudinal Study on Aging, using a convolutional neural network-based algorithm implementing the 2019 ATS/ERS quality criteria [30]. Automated reassessment classified 69.2% of sessions as acceptable and repeatable for both FEV_1_ and FVC, compared with 94.7% classified as acceptable by the original spirometer software. Notably, the entire dataset was processed in less than 10 min, which illustrates the potential for automated quality assessment at scale. However, evidence supporting broader implementation remains limited. A recent systematic review identified only six studies of AI-based spirometry quality control, four evaluating acceptability or usability and two examining the detection of performance errors [24]. Although reported performance was generally promising, with area under the curves (AUCs) ranging from 0.88 to 0.98 among studies reporting this metric, only two studies explicitly used spirometry data obtained in PHC, highlighting the limited external and setting-specific validation of current approaches. Together, these studies demonstrate the progression of AI-based quality control from automated classification of individual manoeuvres to large-scale quality assessment, with evidence that AI-assisted feedback may also improve spirometry acquisition. Such applications are particularly relevant to PHC settings where access to specialist quality review may be limited.

#### 3.2.3. Computational Approaches to Small Airway Dysfunction Detection

The application of AI and machine learning to the detection of SAD remains an emerging area. Bodduluri and colleagues provided early evidence that machine learning can extract information relevant to SAD directly from spirometric flow–volume curves [31]. Using data from 8980 current and former smokers in the COPDGene cohort, the investigators trained a fully convolutional neural network that applied learned filters directly to the sequence of flow values from expiratory flow–volume curves to extract structural patterns associated with CT-defined COPD phenotypes, including functional small airway disease (fSAD) quantified by parametric response mapping [31]. In the held-out test dataset, the neural network achieved an overall AUC of 0.91 (95% CI 0.90–0.92) for phenotyping emphysema and fSAD, compared with 0.80 (95% CI 0.78–0.82) for FEV_1_/FVC and 0.83 (95% CI 0.80–0.84) for FEV_1_ % predicted. For the predominant fSAD phenotype specifically, the neural network achieved an AUC of 0.77 (95% CI 0.73–0.80), compared with 0.58 (95% CI 0.53–0.61) for FEV_1_/FVC and 0.61 (95% CI 0.57–0.64) for FEV_1_ % predicted [31].

Other computational approaches have examined whether clinically relevant information can be derived from expiratory curve morphology. Chen and colleagues developed an automated measure of late-expiratory flattening in patients with preserved FEV_1_/FVC, generating an AB–BC angle (∠ABC) that showed 80.2% concordance with expert identification of this pattern [32]. Providing this quantitative index to eight pulmonologists improved inter-rater agreement from a Fleiss’ κ of 0.313 to 0.515 [32]. Importantly, however, concordance between the curve-derived measure and CT evidence of SAD was only 47.6%, indicating that improved quantification of curve morphology does not necessarily translate into accurate identification of underlying SAD. Nevertheless, the study illustrates how computational analysis can extract and standardise morphological information from the flow–volume curve that would otherwise rely on subjective visual interpretation. In a different application of curve modelling, Maldonado-Franco and colleagues mathematically modelled expiratory flow–volume curves and used the resulting curve coefficients as inputs to an artificial neural network that learned non-linear relationships between curve shape features and diagnostic outcomes through multiple processing layers [33]. In 695 patients undergoing evaluation for suspected respiratory disease, an artificial neural network using coefficients derived from quadratic modelling of the post-bronchodilator curve achieved 92.9% accuracy, 88.2% sensitivity, and 94.3% specificity for COPD classification against expert pulmonologist assessment [33]. Although SAD was not directly assessed, the study provides further evidence that disease-related information can be extracted computationally from expiratory curve morphology, supporting investigation of whether similar approaches could identify features specifically associated with SAD. These findings establish curve morphology as one potential source of information for computational small airway assessment, but alternative approaches have explored whether combining multiple physiological features can further improve classification.

The potential of multi-feature modelling has also been examined using physiological measurements associated with small airway function. In 280 individuals with chronic respiratory symptoms and preserved pulmonary function, supervised machine-learning models incorporating impulse oscillometry achieved substantially greater discrimination for SAD than individual oscillometric parameters [34]. Random forest and AdaBoost, an ensemble method that combines multiple classifiers sequentially giving greater weight to previously misclassified cases, yielded AUCs of 0.914 and 0.915, respectively, compared with 0.642 for R5, the best-performing individual parameter (*p* < 0.01) [34]. More directly relevant to MMEF, Koca evaluated tree-based machine-learning and deep-learning models including convolutional neural networks using Global Lung Function Initiative (GLI)-2012-standardised spirometric features that included FEV_1_, FVC, FEV_1_/FVC, and MMEF [35]. Tree-based models achieved the highest classification performance, with feature-importance analysis identifying MMEF as a contributing feature alongside conventional spirometric and lung-volume variables. Its independent incremental contribution, however, was not evaluated. Collectively, the available evidence supports two complementary computational approaches relevant to small airway assessment: extracting information from expiratory curve morphology and integrating multiple physiological features within machine-learning models. What remains unresolved is whether MMEF contributes clinically meaningful information beyond conventional spirometric and clinical variables. The key studies supporting the current evidence base for AI-assisted spirometry interpretation, quality control, and small airway assessment, selected in accordance with the inclusion and exclusion criteria described in Section 2.3, are summarised in Table 1.

## 4. Discussion

### 4.1. The Opportunity for AI-Enhanced MMEF Interpretation

#### 4.1.1. From MMEF Reporting to Clinical Interpretation

MMEF is commonly reported alongside conventional spirometric indices, yet its clinical interpretation remains poorly standardised. Fixed percentage-of-predicted thresholds are particularly problematic because they do not adequately account for the effects of age, sex, height, and other determinants of expected lung function. In a real-world clinical population, use of an MMEF threshold of <80% predicted produced false-positive rates exceeding 24%, with systematic variation across demographic groups [23]. Expressing MMEF relative to appropriate reference equations using z-scores provides a more statistically appropriate approach to defining abnormality and is consistent with the reference-based interpretation used for conventional spirometric indices [23,36,37]. However, defining an abnormal MMEF does not itself establish its clinical significance or determine what action should follow. This distinction represents an important gap between reporting and clinical interpretation.

Evidence increasingly suggests that reduced MMEF may carry clinically relevant physiological and prognostic information not captured by the presence or absence of conventional airflow obstruction. In the SPIROMICS cohort, lower MMEF was associated with greater disease severity and radiographic emphysema, supporting a relationship between mid-expiratory flow and established COPD phenotype [19]. Longitudinal studies further suggest that abnormal MMEF may precede conventional airflow obstruction. Among 307 patients with normal lung function followed by Kwon and colleagues, COPD developed in 41.8% of those with low baseline MMEF compared with 7.4% of those with preserved MMEF (*p* < 0.001), with low MMEF independently associated with subsequent COPD development (HR 3.31, *p* < 0.001) [16]. Similarly, among smokers without COPD studied by Bazzan and colleagues, 21% of those with MMEF < 80% developed COPD during follow-up compared with 2.7% of those with preserved MMEF (*p* = 0.0004) [17]. In the multinational BOLD study, isolated MMEF below the lower limit of normal in individuals with preserved FEV_1_/FVC was associated with increased odds of developing chronic airflow obstruction over a median follow-up of 8.3 years (OR 2.95, 95% CI 1.02–8.54), with the association replicated in the UK Biobank [38]. In alpha-1 antitrypsin deficiency, individuals with preserved FEV_1_/FVC but reduced MMEF subsequently experienced greater FEV_1_ decline than those with preserved MMEF (−1.09% versus −0.04% predicted per year; *p* = 0.007) [18]. More broadly, a 15-year prospective population-based study using a composite spirometric definition of SAD based on MMEF, FEF_50_ and FEF_75_ found that SAD was associated with accelerated lung function decline and incident COPD [39]. Collectively, these findings suggest that MMEF abnormalities may contain physiological and prognostic information before conventional airflow obstruction becomes apparent. However, they do not establish a single MMEF threshold that should trigger a specific clinical intervention or determine how an abnormal MMEF should be interpreted alongside conventional spirometry and clinical characteristics. This unresolved contextual interpretation, rather than identification of abnormality alone, represents the opportunity for AI-assisted interpretation explored in this review. The three evidence domains summarised in Table 1 each contribute a distinct component of this proposed framework: diagnostic classification models establish the interpretive context within which MMEF would be assessed; quality control models determine whether an individual MMEF value is technically reliable enough to interpret; and computational small airway assessment studies provide proof of concept that information relevant to small airway dysfunction can be extracted from spirometric data, collectively supporting the feasibility of the contextual integration approach proposed here.

#### 4.1.2. How AI Could Enhance MMEF Interpretation

The potential value of AI lies not in interpreting MMEF as an isolated measurement but in integrating it with other spirometric and clinical information to provide an individualised assessment. AI models could incorporate MMEF expressed as a z-score alongside FEV_1_, FVC, FEV_1_/FVC, flow–volume curve morphology, and relevant clinical characteristics. Rather than applying a single threshold to MMEF, a model could identify reduced MMEF with preserved FEV_1_/FVC and estimate its significance in the context of age, smoking or other exposure history, respiratory symptoms, and longitudinal lung function. Existing computational and machine-learning studies provide proof of concept that information relevant to SAD can be extracted from spirometric curves and integrated with multiple physiological features [31,32,34,35], but the incremental value of MMEF within such models has not yet been established. A clinically useful model would therefore need to demonstrate that incorporating MMEF improves prediction or risk stratification beyond conventional spirometric and clinical variables. Such risk stratification could potentially identify a window for earlier preventive action among individuals with preserved conventional spirometry but evidence of increased future respiratory risk. In appropriately selected individuals, this could reinforce smoking cessation and other risk-factor modification, or prompt closer longitudinal surveillance, although whether intervention at this stage alters subsequent disease progression remains unknown.

AI could also translate this integrated assessment into clinically relevant next steps. One potential application is identifying patients with preserved pre-bronchodilator FEV_1_/FVC who nevertheless warrant post-bronchodilator testing. GOLD 2026 recognises the volume-responder phenotype, in which gas trapping reduces FVC and results in a pre-bronchodilator FEV_1_/FVC ≥ 0.70, while a greater increase in FVC after bronchodilation subsequently reveals airflow obstruction [1]. Future models could test whether MMEF, curve morphology, conventional spirometric indices, symptoms, and exposure history together improve prediction of this phenotype and identify patients who would benefit from post-bronchodilator assessment. AI-based quality control could provide an additional safeguard by determining whether an MMEF value is sufficiently reliable to interpret. Because MMEF is particularly sensitive to the quality and completeness of the forced expiratory manoeuvre [12,13], parameter-specific quality assessment could allow for an AI system to flag or suppress MMEF interpretation when technical quality is inadequate. Deep-learning models have already demonstrated the ability to assess spirometric manoeuvre acceptability and usability from spirometric curves [28,29], although MMEF-specific quality assessment has not yet been validated. A conceptual framework for integrating MMEF into AI-assisted spirometry interpretation is presented in Figure 1. 

### 4.2. Challenges and Limitations

#### 4.2.1. Variability and FVC-Dependence of MMEF

A key technical consideration in incorporating MMEF into AI-assisted interpretation is its greater variability and dependence on FVC [13,40]. Because MMEF is calculated over the middle 50% of the exhaled FVC, changes in FVC alter the lung-volume range over which flow is measured and can therefore influence MMEF independently of changes in airway function. MMEF also exhibits greater variability than conventional spirometric indices such as FEV_1_, limiting the reliability of interpretation based on a single measurement [13,40]. These characteristics do not preclude its incorporation into AI models, but they argue against interpreting MMEF in isolation. Models incorporating MMEF should account for manoeuvre quality, FVC, curve morphology, and potentially longitudinal measurements when estimating the significance of an abnormal value. Approaches that adjust MMEF for changes in FVC have been proposed to address FVC dependence [41,42], although these methods are not currently part of routine spirometric interpretation.

#### 4.2.2. Absence of Validated Clinical Decision Thresholds

A more fundamental limitation is the absence of validated clinical decision thresholds for MMEF [12]. A z-score below the lower limit of normal identifies a statistically abnormal value but does not establish the probability of future disease or specify what clinical action should follow. Similarly, no validated minimal clinically important difference has been established for longitudinal changes in MMEF [43]. Without validated thresholds linking the degree of MMEF abnormality to specific clinical outcomes and actions, increased sensitivity could also lead to overinterpretation, unnecessary investigation, or inappropriate disease labelling. This distinction is important for AI-assisted interpretation: identifying abnormality is considerably simpler than determining its clinical significance. However, AI models trained on sufficiently large longitudinal datasets could potentially estimate outcome-based risk associated with MMEF across a continuous range of values, rather than relying on a single predetermined threshold. Such models would require prospective validation against clinically relevant outcomes before their predictions could inform patient management.

#### 4.2.3. Generalisability and Reference Equation Limitations

Generalisability represents a broader challenge for both AI-based respiratory diagnostics and MMEF itself. Existing AI approaches differ substantially in training populations, input modalities, clinical settings, and validation strategies, and performance cannot be assumed to transfer unchanged across populations, healthcare systems, devices, and patterns of respiratory disease exposure [24,44]. A recent systematic review and meta-analysis of 56 deep-learning studies for COPD diagnosis found promising performance for binary disease detection but substantial between-study heterogeneity and limited external validation, highlighting the need for reproducible multicentre validation across diverse populations and healthcare settings [44]. Reference equations introduce an additional consideration for MMEF. Although the GLI Global 2022 equations provide a race-neutral approach for FEV_1_, FVC, and FEV_1_/FVC, equivalent global equations are not currently available for MMEF [45]. AI models incorporating MMEF z-scores would therefore need to account explicitly for the reference equations used and should be externally validated across diverse populations. Development of population-inclusive reference standards for MMEF would strengthen the basis for future AI-assisted interpretation.

#### 4.2.4. Regulatory and Workflow Integration Barriers

Even a well-validated AI model would require integration into existing spirometry and clinical workflows. Regulatory approval, interoperability with different spirometry devices and electronic health records, data governance, and requirements for local validation may all influence implementations. Qualitative evidence from PHC further indicates that successful implementation will depend on clinician and commissioner trust, robust validation, and the ability of AI to support existing spirometry quality assessment and interpretation pathways [46]. In interviews with 28 clinicians and commissioners involved in spirometry services in England, AI was generally viewed positively as a potential aid to quality assessment and interpretation, although concerns regarding implementation and reliance on automated outputs were also identified. These concerns are particularly relevant to AI-enhanced MMEF interpretation, where an abnormal value should be contextualised alongside symptoms, exposure history, conventional spirometric indices, and other clinical information rather than treated as an autonomous diagnosis. AI should therefore complement rather than substitute for investment in trained personnel, standardised testing procedures, appropriate equipment, and quality assurance. Implementation studies will be needed to determine whether AI-assisted interpretation improves clinical decision-making without increasing workflow burden or inappropriate downstream testing.

## 5. Research Implications

Several research priorities emerge from this review. The most immediate is the development and validation of AI models that explicitly evaluate the incremental value of MMEF beyond conventional spirometric and clinical variables. Such models should incorporate MMEF as a continuous z-score alongside FEV_1_, FVC, FEV_1_/FVC, flow–volume curve morphology, symptoms, exposure history, and other relevant clinical characteristics, and should be trained against longitudinal outcomes. Particular attention should be given to individuals with reduced MMEF but preserved FEV_1_/FVC, allowing for models to determine whether this pattern independently predicts progression to established airflow obstruction, accelerated lung function decline, or other clinically relevant outcomes. The need for such analysis is illustrated by Wang and colleagues, who developed machine-learning models for accelerated FEV_1_ decline using data from COPDGene with external validation in SPIROMICS [47]. Although MMEF was initially considered as a candidate feature, it was excluded during feature selection because of its strong correlation with FEV_1_/FVC. This approach addresses feature redundancy but does not establish whether MMEF provides incremental prognostic information beyond conventional spirometric indices, particularly among individuals with preserved FEV_1_/FVC. Comparisons of otherwise equivalent models with and without MMEF would therefore be essential to establish its incremental predictive value.

A complementary priority is establishing the clinical significance of longitudinal change in MMEF. Although longitudinal studies have associated reduced MMEF with subsequent airflow obstruction and lung function decline [16,17,18,19], a validated minimal clinically important difference for MMEF is not currently established, limiting interpretation of serial measurements and treatment-related change [43]. Studies relating longitudinal MMEF trajectories to symptoms, health-related quality of life, exacerbations, and other clinically meaningful outcomes could address this gap. A methodological precedent exists for other measures of small airway function. Abdo and colleagues derived minimal clinically important differences for impulse oscillometry measures in adults with asthma and validated these against changes in asthma control and health-related quality of life [48]. A similar approach could be applied to MMEF to determine whether longitudinal changes correspond to clinically meaningful outcomes.

If incremental predictive value is demonstrated, prospective studies should then determine whether providing MMEF-enhanced AI output changes clinical decision-making and ultimately improves patient outcomes. The SPIRO-AID trial provides a methodological precedent for evaluating AI-assisted spirometry interpretation in PHC [15]. Future trials could compare conventional spirometry interpretation, AI assistance without MMEF-specific information, and AI assistance incorporating MMEF to determine its incremental effect on appropriate post-bronchodilator testing, specialist referral, diagnostic accuracy, and longitudinal clinical outcomes. Importantly, studies should also quantify potential harms, including unnecessary investigation, referral, and disease labelling.

Finally, models should undergo external validation across diverse PHC populations, healthcare systems, spirometry devices, and demographic groups before widespread implementation [24,44]. Development of globally applicable reference equations for MMEF, analogous to the race-neutral GLI Global equations available for conventional spirometric indices, would further strengthen the basis for equitable MMEF interpretation [45]. Reporting model performance across relevant demographic and clinical subgroups will also be essential to identify potential algorithmic bias before deployment.

## 6. Conclusions

In conclusion, spirometry underuse and interpretive limitations in PHC highlight the potential role of AI-assisted approaches beyond conventional spirometric interpretation. Current evidence suggests that reduced MMEF, particularly in the presence of preserved FEV_1_/FVC, may provide clinically relevant information before conventional airflow obstruction becomes apparent. AI-assisted approaches may facilitate the integration of MMEF with other spirometric and clinical information; however, the incremental predictive value and clinical utility within such approaches remain uncertain. Further studies are needed to validate these approaches and determine their potential contribution to risk assessment and clinical decision-making.

## Figures and Tables

**Figure 1:**
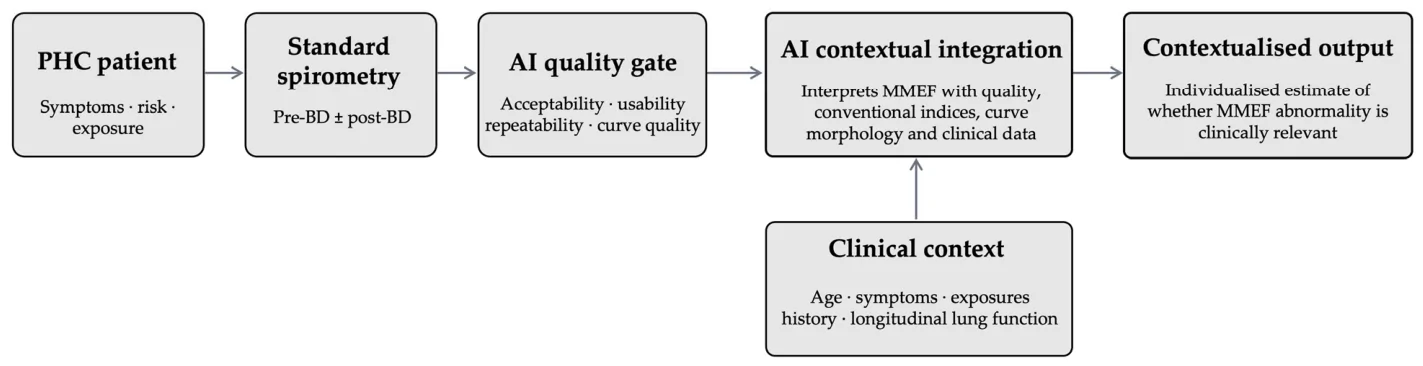
Proposed framework for AI-enhanced interpretation of MMEF in primary care spirometry. Following AI-assisted assessment of spirometry quality, MMEF is interpreted in the context of conventional spirometric indices, flow–volume curve morphology, and relevant clinical information rather than as an isolated parameter. The resulting output represents an individualised estimate of the clinical relevance of an MMEF abnormality. This conceptual framework illustrates a potential application of AI-assisted spirometry and requires prospective validation before clinical implementation. AI, artificial intelligence; BD, bronchodilator; MMEF, maximal mid-expiratory flow; PHC, primary healthcare.

**Table 1:** Key studies of artificial intelligence in spirometry interpretation, quality control, and small airway assessment relevant to AI-enhanced MMEF interpretation.

Evidence Domain	Study	AI Approach	Input Data	Primary Task	Performance (Key Metric)	MMEF/SAD Relevance	PHC Evaluation
Diagnostic interpretation	Topalovic et al. [25]	Machine learning (supervised)	Full PFT data	Diagnostic classification	82% accuracy vs. 44.6% pulmonologists	None	No
	Das et al. [26]	Explainable AI (XAI)	Full PFT data	Diagnostic classification with explanation	Accuracy improved from 69.3% to 74.6% with AI + clinician	None	No
	Sunjaya et al. [27]	Random forest	Raw spirometry + demographics	COPD/asthma/ILD classification	AUC 0.914 (COPD); sensitivity 84.0%, specificity 86.8%	None	Yes
	Doe et al. SPIRO-AID [15]	AI decision support (RCT)	Spirometry records	Clinician interpretation support	+9.0 pp diagnosis accuracy; +15.9 pp COPD subgroup (*p* < 0.001)	None	Yes
	Maldonado-Franco et al. [33]	ANN with mathematical curve modelling	Pre-/post-BD expiratory flow–volume curves (*n* = 695)	COPD classification from curve morphology	92.9% accuracy; 88.2% sensitivity; 94.3% specificity	None; curve morphology analysed	No
Quality control	Das et al. [28]	Convolutional neural network	Spirometry curves (*n* = 36,873)	Manoeuvre quality control	87% acceptability, 92% usability accuracy	None; general spirometry quality	No
	Wang et al. [29]	Deep learning	Spirometry files (*n* = 16,502)	Quality grading + clinician feedback	95.1% FEV1 acceptability; +21–36% good-quality tests in GPs	None; general spirometry quality	GP setting
	Bowerman et al. [30]	CNN-based automated quality-control algorithm	Population spirometry (*n* = 21,795)	Population-scale spirometry quality assessment	69.2% classified acceptable and repeatable for both FEV_1_ and FVC; entire dataset processed in <10 min	None; general spirometry quality	No; population cohort
Small airway assessment	Bodduluri et al. [31]	Convolutional neural network	Flow–volume curves (COPDGene, *n* = 8980)	CT-defined COPD phenotyping including fSAD	AUC 0.91 overall; AUC 0.77 for fSAD phenotype vs. 0.58 for FEV1/FVC	fSAD predicted from spirometric curves; MMEF not evaluated	No
	Chen et al. [32]	Automated mathematical curve modelling	Flow–volume loops	Quantification of late-expiratory flattening associated with SAD	∠ABC: 80.2% concordance with expert identification; κ improved from 0.313 to 0.515; 47.6% concordance with CT evidence of SAD	SAD-related curve morphology; MMEF not evaluated	No
	Xu et al. [34]	Random forest, AdaBoost	Impulse oscillometry (*n* = 280)	SAD detection in preserved spirometry	AUC 0.914–0.915 vs. AUC 0.642 for best single IOS parameter	SAD detection using IOS; MMEF not evaluated	No
	Koca [35]	Tree-based ML, deep learning	GLI-2012 spirometry features including MMEF	Pulmonary disease classification	Best performance with tree-based models; MMEF included as feature	MMEF included; incremental value not isolated	No

Study inclusion and exclusion criteria are described in Section 2.3. AI, artificial intelligence; ANN, artificial neural network; AUC, area under the receiver operating characteristic curve; COPD, chronic obstructive pulmonary disease; CT, computed tomography; FEF_25–75_, forced expiratory flow between 25% and 75% of forced vital capacity; FEV_1_, forced expiratory volume in one second; fSAD, functional small airway disease; FVC, forced vital capacity; GLI, Global Lung Function Initiative; GP, general practitioner; ILD, interstitial lung disease; IOS, impulse oscillometry; ML, machine learning; MMEF, maximal mid-expiratory flow; PFT, pulmonary function test; PHC, primary healthcare; pp, percentage points; RCT, randomised controlled trial; SAD, small airway dysfunction; XAI, explainable artificial intelligence.

## Data Availability

No new data were created or analyzed in this study. Data sharing is not applicable to this article.

## Abbreviations

The following abbreviations are used in this manuscript:

AI	Artificial intelligence
ATS	American Thoracic Society
AUC	Area under the curve
COPD	Chronic obstructive pulmonary disease
CT	Computed tomography
ERS	European Respiratory Society
FEF_25–75_%	Forced expiratory flow between 25% and 75% of forced vital capacity
FEV_1_	Forced expiratory volume in one second
fSAD	Functional small airway disease
FVC	Forced vital capacity
GLI	Global Lung Function Initiative
MMEF	Maximal mid-expiratory flow
PHC	Primary healthcare
RCT	Randomised controlled trial
SAD	Small airway dysfunction
